# Microbial Regulation in Gorgonian Corals 

**DOI:** 10.3390/md10061225

**Published:** 2012-06-04

**Authors:** Laura R. Hunt, Stephanie M. Smith, Kelsey R. Downum, Laura D. Mydlarz

**Affiliations:** 1 Department of Biology, University of Texas at Arlington, 235 Life Science, Arlington, TX 76019, USA; Email: hunt.laura@epa.gov (L.R.H.); crader@wisc.edu (S.M.S.); downumk@uta.edu (K.R.D.); 2 United States Environmental Protection Agency Region 6, 1445 Ross Avenue, Dallas, TX 75202, USA

**Keywords:** gorgonian corals, quorum sensing (QS), antimicrobial activity, microbial regulation

## Abstract

Gorgonian corals possess many novel natural products that could potentially mediate coral-bacterial interactions. Since many bacteria use quorum sensing (QS) signals to facilitate colonization of host organisms, regulation of prokaryotic cell-to-cell communication may represent an important bacterial control mechanism. In the present study, we examined extracts of twelve species of Caribbean gorgonian corals, for mechanisms that regulate microbial colonization, such as antibacterial activity and QS regulatory activity. Ethanol extracts of gorgonians collected from Puerto Rico and the Florida Keys showed a range of both antibacterial and QS activities using a specific *Pseudomonas aeruginosa* QS reporter, sensitive to long chain AHLs and a short chain *N*-acylhomoserine lactones (AHL) biosensor, *Chromobacterium violaceium*. Overall, the gorgonian corals had higher antimicrobial activity against non-marine strains when compared to marine strains. *Pseudopterogorgia americana*, *Pseusopterogorgia acerosa*, and *Pseudoplexuara flexuosa* had the highest QS inhibitory effect. Interestingly, *Pseudoplexuara porosa* extracts stimulated QS activity with a striking 17-fold increase in signal. The stimulation of QS by *P. porosa* or other elements of the holobiont may encourage colonization or recruitment of specific microbial species. Overall, these results suggest the presence of novel stimulatory QS, inhibitory QS and bactericidal compounds in gorgonian corals. A better understanding of these compounds may reveal insight into coral-microbial ecology and whether a therapeutic potential exists.

## 1. Introduction

Gorgonian corals are found in coral reefs worldwide, but are most abundant and diverse in Caribbean reefs [1]. The Caribbean gorgonians are dominant over scleractinian corals—both in size and number [1,2], are a rich source of natural products [3], and are important ecological components in Caribbean reef habitats [4,5]. Their success can be attributed partly to their diverse array of natural products that act as feeding deterrents against fish and invertebrate predators [3,6,7]. In addition, gorgonian natural products may also play a role in antifouling [8] and in mediating coral-microbe interactions [9,10,11]. Microbes are abundant in marine systems and natural constituents of healthy corals including a surface mucus layer [12,13,14,15]. It appears that many corals host rich bacterial diversity that is distinct from the water column, species specific, and play protective roles in the coral “holobiont” (symbiotic organism that includes the coral animal polyp, endosymbiotic dinoflagellate, and bacteria [16,17,18,19]). However, when coral bacteria communities are disrupted, this may render corals more susceptible to disease and mortality from pathogenic bacteria [11,20,21]. Thus, keeping microbial populations under control is a very important mechanism to maintaining coral health. 

Corals may control microbe populations with immune defenses [22,23], self-cleaning of mucus [24], or secrete chemicals that inhibit microbial growth, attachment, and behavior [8,11,25]. Such chemicals could include compounds that regulate bacteria communication or have antimicrobial activity. Bacteria communicate with one another using signaling molecules which accumulate in the environment with increasing bacterial density in a process termed quorum sensing (QS). When the concentration of signaling molecules accrues beyond a certain threshold, they interact with their cognate receptor and upregulate target gene expression. The best characterized QS molecules are *N*-acylhomoserine lactones (AHL) [26,27,28] produced by Gram-negative bacteria. Due to the prevalence of Gram-negative bacteria in the marine environment, we focused on AHL-based QS signaling for this study. Gram-negative bacteria use QS to facilitate and coordinate a range of cellular processes such as motility [29], luminescence [30], biofilm formation [31], expression of virulence factors [32], and is an important mechanism in the colonization of eukaryotic organisms [33,34]. Growing documentation of QS activity in corals, suggest that QS is also important in coral-bacteria associations. Extracts of various stony and soft corals from the Great Barrier Reef have QS regulatory properties [35,36] and recently, production of QS signals have been reported in cultures of coral associated bacteria [37,38]. Most notable, Alagely *et al*. [39] demonstrated that compounds isolated from the surface of healthy corals could modulate QS regulated behavior in the coral pathogen *Serratia marcescens*. 

In the Caribbean, different studies have produced conflicting conclusions as to whether gorgonians possess compounds with antibacterial activity against marine bacteria and whether this is a significant component of microbial defense [9,10]. The differences between studies are mostly attributed to different interpretations of what constitutes significant antimicrobial activity based on the disc diffusion assay method. Furthermore, Jensen *et al*. [9] suggest that the maintenance of broad-spectrum antimicrobial activity is not a leading factor in the evolution of chemical defenses in gorgonians. In addition to revisiting the scope of antibacterial activity in corals, it is also necessary to address other mechanisms for microbial regulation in gorgonians. The intricacy of how gorgonians regulate microbial populations remains largely unknown. It is not clear whether gorgonians have intrinsic chemical defenses against potentially pathogenic marine bacteria or other mechanisms for microbial regulation, such as quorum sensing (QS). Caribbean gorgonians have properties that modulate bacterial physiology and quorum sensing (QS) has been suggested as a possible mechanism [11]. Yet, to date, Caribbean gorgonians have not been surveyed for QS activity. The overall objective of this study was to determine whether or not Caribbean gorgonians have detectable levels of antimicrobial and QS activity. We surveyed dominant Caribbean gorgonians for the presence of QS activity and bactericidal activity against a suite of model bacterial strains, including ecologically relevant marine bacteria known to cause coral diseases. The gorgonians studied represent dominant genera from two locations in the Caribbean, Puerto Rico and the Florida Keys. We hypothesized that antimicrobial and QS activity would be detectable but would vary among the dominant gorgonian genera in the Caribbean.

## 2. Results

### 2.1. Antimicrobial Activity

**Figure 1:**
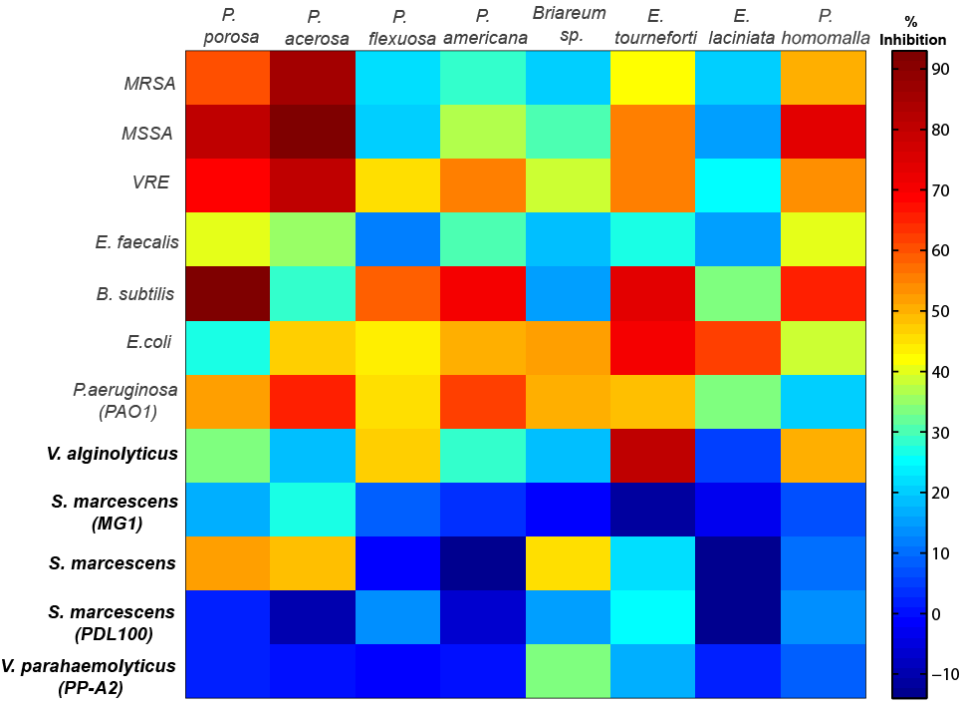
Cell plot with % growth inhibition of various gorgonian coral extracts against non-marine and marine bacteria (bold) strains. The red end of the spectrum represents very high bactericidal activity while the blue end of the spectrum represents little to no inhibitory activity.

**Figure 2:**
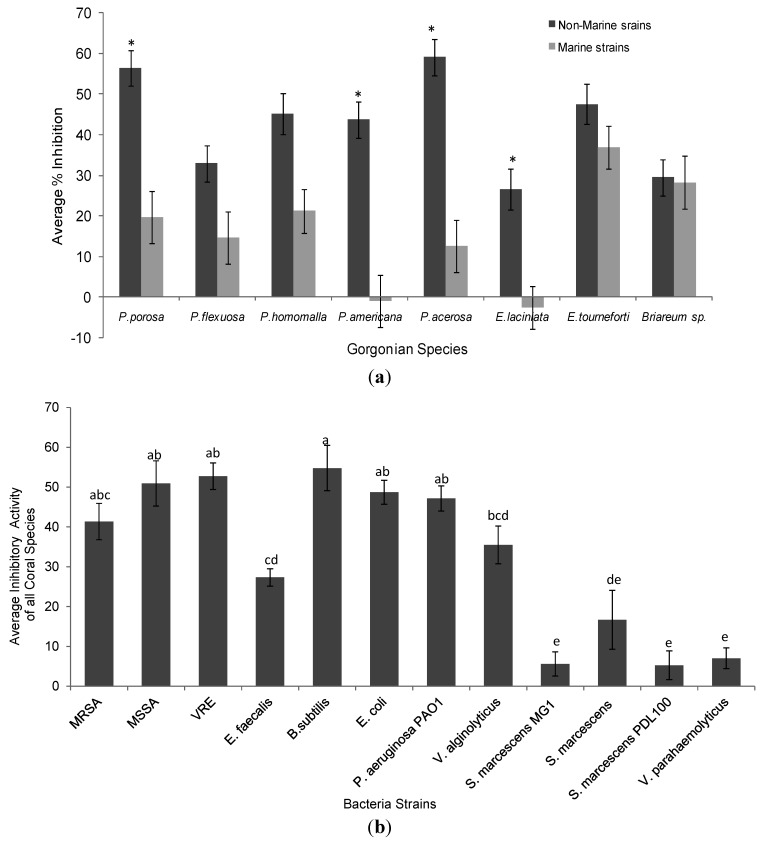
(**a**) Average % growth inhibition of gorgonian coral extracts against non-marine and marine strains of bacteria. Data represents mean ± standard error. Asterisks indicate statistical difference in growth inhibition between non-marine and marine strains of the same coral extracts at *p* < 0.05 using Tukey post-hoc test; (**b**) Average % growth inhibition of all gorgonian corals against each tester strain of bacteria. Data represents mean ± standard error. Letters indicate statistical difference between inhibition of bacteria strains at *p* < 0.05 using Tukey post-hoc test. % growth inhibitions labeled with (e) do not differ significantly from the controls or zero.

Ethanol extracts from all eight gorgonians showed significant but variable antibacterial activity against Gram-negative and Gram-positive bacterial strains (Table 1, Table 2 and Figure 1). Overall the extracts from *Pseudoplexaura porosa* and *Pseudopterogorgia acerosa* demonstrated the highest inhibitory activity against all the bacteria strains, and *Eunicea laciniata* had the lowest bactericidal activity. Gorgonian extracts had greater inhibitory activity against Gram-positive than Gram-negative bacteria (Table 2). *P. porosa*, *Plexaura homomalla*, *Pseudopterogorgia americana* and* P. acerosa* all demonstrated significantly higher activity against Gram-positive strains. The gorgonian coral extracts also exhibited significantly higher bactericidal activity against non-marine compared to marine strains (Figure 2a). *P. porosa*, *P. Americana*, *P. acerosa* and *E. laciniata* had higher activity against non-marine bacterial strains, while *Briareum* sp. had the same activity against all bacteria regardless of the Gram staining or environmental source. 

**Table 1:** Description of marine and non-marine bacteria used in antimicrobial bioassays.

Bacteria species	Environment	Gram Stain	Source
Methicillin resistant *Staphylococcus aureus* (MRSA)	Non marine	+	ATCC # 29213
Methicillin sensitive *Staphylococcus aureus* (MSSA)	Non marine	+	ATCC # 43300
Vancomycin resistant *Enterococcus faecium* (VRE)	Non marine	+	ATCC # 700221
*Enterococcus faecalis*	Non marine	+	ATCC # 29212
*Bacillus subtilis*	Non marine	+	ATCC # 6051
*Escherichia coli*	Non marine	−	ATCC # 10536
*Pseudomonas aeruginosa* (PAO1)	Non marine	−	ATCC # 39018
*Serratia marcescens* (MG1)	Non marine	−	M. Teplitski
*Serratia marcescens*	Marine	−	ATCC # 39006
*Serratia marcescens* (PDL100)	Marine	−	ATCC # BAA-632
*Vibrio alginolyticus*	Marine	−	GenBank # X744690
*Vibrio parahaemolyticus* (PP-A2)	Marine	−	Genbank # FJ892748

**Table 2:** ANOVA statistical data for antibacterial activity.

Factor	ANOVA data		
	*F*	df	*p*
Coral species	63.50	7	<0.0001
Bacteria species	153.65	11	<0.0001
Coral species × Bacteria species	16.87	77	<0.0001
Gram stain (+) or (−)	14.71	1	0.0002
Coral species × Gram stain	5.11	7	<0.0001
Environment (non-marine or marine)	14.74	1	0.0002
Coral species × Environment	4.41	7	<0.0001

Among the bacteria strains, *Bacillus subtilis* was most sensitive to gorgonian extracts followed by vancomycin resistant *Enterococcus* (VRE) and methicillin-sensitive *Staphylcoccus aureus* (MSSA), *Escherichia coli* and* Pseudomonas aeruginosa* strain PAO1 (Table 2 and Figure 2b). The coral extracts had higher inhibitory activity against the vacomycin resistant Enterococcus than the susceptible strain, while there was no difference in inhibitory activity between the drug resistant *Staphylcoccus aureus* and the resistant strain. In contrast, very little inhibitory activity was observed against many of the marine bacteria, in fact there was negligible activity against all three *S. marcescens* strains (not different from the control). Of the *Vibrio* species, there was higher susceptibility to the coral extracts in *V. alginolyticus* than *V. parahaemolyticus*. 

### 2.2. Quorum Sensing (QS) Inhibitory Activity in Gorgonian Extracts

**Figure 3:**
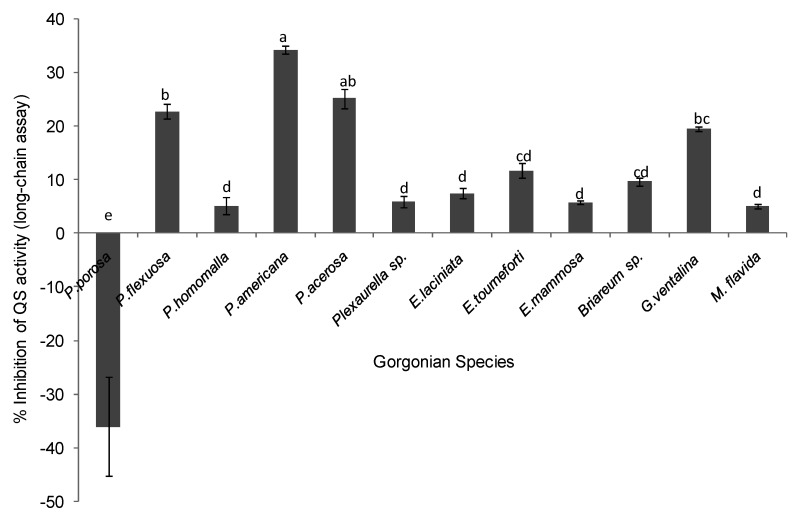
Inhibition of quorum sensing (QS) by gorgonian extracts in the long chain acyl homoserine lactone (AHL) bioassay. Values represent % QS inhibition compared to vehicle control, which was set to 100% (*n* = 3 replicate wells, ANOVA *F* = 53.39, *p* < 0.0001). Data represents mean ± standard error. Letters indicate statistical difference at *p* < 0.05 using Tukey post-hoc test between coral species, although those labeled with (d) or (cd) were not different from the control or zero and thus are not considered to be inhibitory.

The potential of gorgonian extracts to antagonize bacteria cell-cell communication systems was investigated using two reporter bioassays, *Pseudomonas aerugionosa* 3-oxo-C12-HSL (long chain AHL) and *Chromobacterium violaceum* CV026 (short chain AHL)*.* Differences in antagonistic effects in the long chain AHL assay were observed among the corals tested (ANOVA *F* = 53.39, *p* < 0.0001, Figure 3). The gorgonians *Pseudopterogorgia americana*, *Pseudopterogorgia acerosa*, and *Plexuara flexuosa* and *Gorgonia ventalina* had the highest inhibitory effect on QS, while the other coral species did not have significant inhibitory activity above the controls. To exclude the possibility that reduced fluorescence was a result of growth inhibition rather than antagonism of QS activity, growth curves of *P. aeruginosa* PAO1-JP2 (pKR-C12) with and without extracts were determined. At a concentration of 250 µg/mL, extracts did not significantly affect bacterial growth of the las/rhl1 double mutant PAO1-JP2 reporter strain with pKR-C12 biosensor during the incubation time.

**Figure 4:**
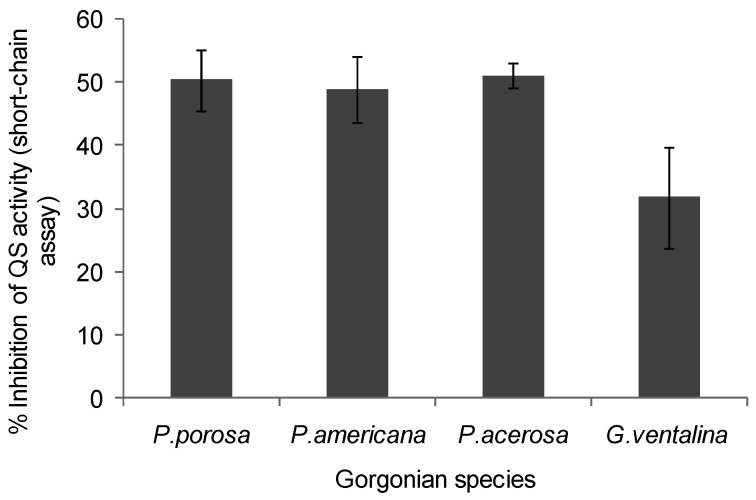
Inhibition of violacein production by *Chromobacterium violaceum* CV026 by gorgonian extracts. Values represent % quorum sensing (QS) inhibition compared to vehicle control, which was set to 100% (*n* = 3 replicate wells, ANOVA *F* = 29.03, *p* < 0.0001). Data represents mean ± standard error. Only the gorgonians, *P. porosa*, *P. americana*, *P. acerosa* and *G. ventalina* had detectable inhibitory activity, although here was no discernible differences between the corals using Tukey post-hoc test.

A decrease in violacein was expected in the *Chromobacterium violaceum* CV026 assay if coral extracts contained anti-QS compounds that interfered with short chain AHLs or their production. Violacein production was reduced by 50% in the presence of *Pseudopterogorgia acerosa*, *Pseudopterogorgia americana*, and *Pseudoplexaura porosa* extracts, while *Gorgonia ventalina* inhibited violacein by 30% (ANOVA *F* = 29.03, *p* < 0.0001, Figure 4). All other gorgonians tested did not affect violacein production in the *Chromobacterium violaceum* CV026 bioassay. Growth inhibition was also tested for *C. violaceum*, but none of the extracts affected bacterial growth (data not shown).

### 2.3. Quorum Sensing (QS) Stimulatory Activity in Gorgonian Extracts

**Figure 5:**
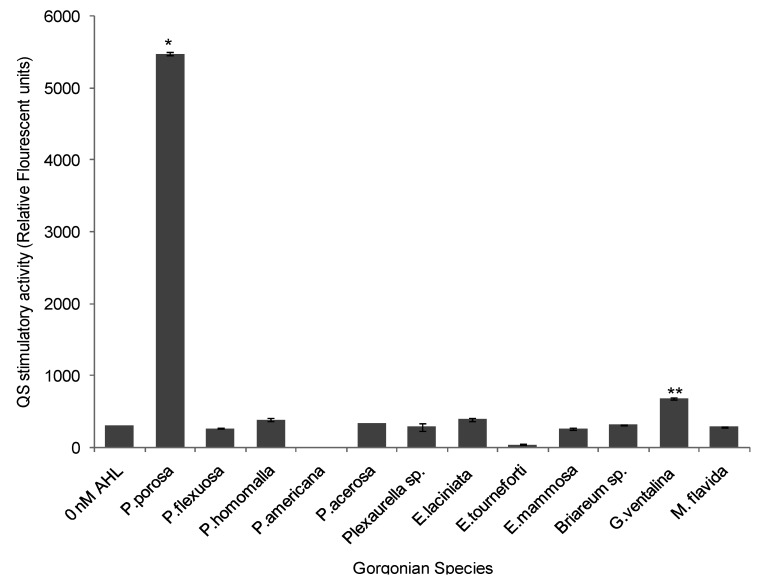
Stimulation of quorum sensing (QS) by various gorgonian extracts in the long chain acyl homoserine lactones (AHL) bioassay. Values were reported as relative fluorescent units (RFU) (*n* = 3 replicate wells, ANOVA *F* = 7314.64, *p* < 0.0001). Data represents mean ± standard error. Asterisks indicate gorgonians, which have greater than 1 fold induction over negative control (without the addition of synthetic AHL (3-oxo-C12-HSL). There was statistical significance at *p* < 0.05 using Tukey post-hoc test between the stimulatory QS activity of *P. porosa* and *G. ventalina*.

With the exception of *Pseudoplexaura porosa* and *Gorgonia ventalina*, none of the gorgonian extracts induced QS activity in the long chain AHL bioassay. *P. porosa* extract stimulated QS activity with a maximum 17-fold increase in signal over negative control (ANOVA *F* = 7314.64, *p* < 0.0001, Figure 5). *G. ventalina* had low, but appreciable, pro-QS activity with a 2-fold induction over negative control. For the *Chromobacterium violaceum* CV026 assay, none of the gorgonian extracts induced violacein production.

## 3. Discussion

Antimicrobial and quorum sensing (QS) compounds have been proposed as potential mechanisms for microbial regulation in Caribbean corals [9,10,11,37,38,39,40]. Overall, our study is in agreement with Kim 1994 [10] and Jensen *et al*. 1996 [9] in that the incidence of antibiotic activity against non-marine bacteria was significantly greater than marine strains. Specifically, we detected little activity against all three *S. marcescens* strains and *Vibrio parahaemolyticus* PP-A2, a strain isolated from the surface of healthy *Pseudoplexaura porosa*. *S. marcescens* PDL100 and *V. alginolyticus* are both coral pathogens, it is possible that these and other marine bacteria are successful pathogens due to the low bactericidal activity or resistance of the corals and their ethanol extracts. 

In addition, little activity was observed against Gram-negative strains relative to Gram-positive strains of bacteria, which supports the notion that gorgonians lack broad-spectrum antimicrobial activity. Jensen *et al*. [9] has suggested that antimicrobial activity might not be a crucial factor in the evolution of secondary metabolites. This may be a characteristic that is specific to Caribbean gorgonians, because broad-spectrum activity is found in corals from other locations around the globe [41]. Caribbean gorgonians may rely on other mechanisms, such as inducible immune defenses [22,23,42,43] or QS, for microbial regulation. Nonetheless, the presence of strong antimicrobial activity against human pathogens is notable for the potential of development of novel antimicrobial therapies, especially against antibiotic resistant bacterial strains [44]. 

There is growing evidence that bacteria cooperative consortia (known as biofilms) with higher organisms may involve regulation of quorum sensing (QS) [31,45,46,47]. We investigated QS antagonism and inductive activity in Caribbean gorgonian corals as a possible method for microbial control. Considering the widespread occurrence of Gram-negative bacteria in the marine environment [48,49,50] and the prevalence of *N*-acyl-homoserine lactones in marine bacteria [36,51,52], our investigation targeted LuxI/LuxR-type quorum sensing systems of Gram-negative bacteria. We used two different QS reporters for detecting long and short chain AHL signaling molecules in the gorgonian extracts. Our results show that Caribbean gorgonian corals possess compounds that both antagonize and stimulate or induce QS activity (summarized in Table 3). A third of the gorgonians screened had activity that antagonized QS activity, while only two induced QS activity. QS activity seemed to be genus specific, as activity was restricted to corals from the genera *Pseudoplexaura*, *Plexaura*, *Pseudopterogorgia* and *Gorgonia*. The three species from the genus *Eunicea*, along with *Briareum* and *Muriceopsis* lacked any QS inhibitory or inductive activity entirely.

**Table 3:** Summary of inhibitory and inductive quorum sensing (QS) activities of crude ethanol extracts from 12 Caribbean gorgonian corals in *Pseudomonas aeruginosa* long chain acyl homoserine lactone (C12-AHL) and *Chromobacterium violaceum* CV026 short chain (C6-HHL) bioassays. Results are shown as presence (+) or absence (−) of either inhibitory or inductive QS signal.

Collection Location	Coral Species	Inhibitory QS Biossays		Inductive QS Biossays	
		*Pseudomonas aeruginosa* Long chain AHL	*Chromobacterium violaceum* CV026 Short chain AHL	*Pseudomonas aeruginosa* Long chain AHL	*Chromobacterium violaceum* CV026 Short chain AHL
Puerto Rico	*Pseudoplexaura porosa*	−	+	+	−
Puerto Rico	*Plexaura flexuosa*	+	−	−	−
Puerto Rico	*Plexaura homomalla*	−	−	−	−
Puerto Rico	*Pseudopterogorgia americana*	+	+	−	−
Puerto Rico	*Pseudopterogorgia acerosa*	+	+	−	−
Florida Keys	*Plexaurella* sp.	−	−	−	−
Puerto Rico	*Eunicea laciniata*	−	−	−	−
Puerto Rico	*Eunicea tourneforti*	−	−	−	−
Florida Keys	*Eunicea mammosa*	−	−	−	−
Puerto Rico	*Briareum* sp.	−	−	−	−
Puerto Rico	*Gorgonia ventalina*	+	+	+	−
Florida Keys	*Muriceopsis flavida*	−	−	−	−

Moderate quorum sensing (QS) inhibition was detected in *Pseudopterogorgia acerosa*, *Plexuara flexuosa*, *Pseudopterogorgia americana* and *Gorgonia ventalina*, using a specific *Pseudomonas aeruginosa* screen (long chain AHLs), while more potent inhibition was found in *P. americana*, *P. acerosa*, *P. porosa*, and *G. ventalina* using the *Chromobacterium violaceum* screen (short chain AHLs). Interestingly, *P. acerosa* and *P. americana* were antagonistic in both bioassays, which may indicate a mechanism conserved within the genus *Pseudopterogorgia*. The genus *Pseudopterogorgia* is known for having a rich diversity of bioactive natural products with diverse in vitro activities, such as anti-inflammatory [53], antimicrobial components [9,11] and ichthyodeterrents [6,54]. In addition, *P. americana* has copious amounts of mucus with properties that regulate metabolic and antibiotic activity in coral-associated bacteria and was suggested to be under QS control [11]. Skindersoe *et al*. [35] reported that true soft corals (Order Alcyonacea) from the Great Barrier Reef exhibited the highest levels of quorum sensing (QS) inhibitory activity in proportion to other marine organisms examined. The occurrence of QS antagonism in the Great Barrier Reef and Caribbean coral extracts suggests QS activity is a widespread phenomenon in Anthozoan-microbial interactions. 

Eukaryotes and prokaryotes can both activate or inhibit QS, which can elicit a range of functional responses in either group [55,56,57]. For example, bacteria isolated from marine sponges produce AHLs that are hypothesized to promote bacterial colonization of sponge surfaces [36,52]. In contrast, the red alga *Delisea pulchra*, synthesizes AHL mimics, known as furanones, that disrupt QS signaling and inhibit bacterial colonization of the alga surfaces [58,59]. Moreover, resident coral bacteria produce compounds that regulate the growth of other microbes [11] and may also be a source of QS antagonistic compounds. 

It is important to note the limitations of the assays used in this study. The gorgonian extracts may have AHL concentrations below the threshold of sensitivity to the biosensors or nonspecific AHLs to the biosensor. In addition, the long chain AHL *Pseudomonas aeruginosa* biosensor has a limited range of detection for the acyl moiety and is most sensitive to 3-oxo-C12-HSL and 3-oxo-C10-HSL AHLs. 

Strong quorum sensing (QS) induction was detected in *Pseudoplexuara porosa* while very moderate levels of QS stimulation were observed in *Gorgonia ventalina* in the *Pseudomonas aeruginosa* long chain AHL assay*.* In contrast, no QS induction was detected with the *Chromobacterium violaceum* CV026 assay. The ability of *G. ventalina* to have both very moderate anti-QS activity as well as moderate pro-QS activity is very interesting. Since no exogenous AHLs are added in the QS induction assay, it is entirely possible that extracts from *G. ventalina* have several different types of compounds in their crude extracts. Some compounds may mimic AHLs to stimulate QS, and some inhibit the QS activation pathway, or bind to the exogenous AHL to inhibit QS. Since this was not a phenomenon that occurred with any of the other 11 gorgonian corals examined, it is possible this is a phenomenon unique to *G. ventalina*. Of the corals we examined, *G. ventalina* is one of the most studied mostly due to its susceptibility to fungal and parasitic diseases [60]. As such, many different types of defense mechanisms from antifungal to antibacterial defenses have been identified [61,62]. The ability to moderately inhibit and stimulate QS may be a direct or indirect result of the diversity of defensive strategies employed by this coral. 

Crude extracts from *Pseudoplexaura porosa*, on the other hand, very definitively stimulated QS and produced a signal that was comparable to approximately 40% of the inducing synthetic AHL (50 nM, 3-oxo-C12-HSL). Furthermore, *P. porosa* extracts showed high levels of inhibition in the *Chromobacterium violaceum* CV026 bioassay. Long chain AHLs (*N*-acyl side chains from C 10 to C 14), in the presence of *N*-hexanoylhomoserine lactones (HHL) are known to inhibit violacein pigment production in the *Chromobacterium violaceum* CV026 bioassay [63,64]. In our studies, there was a negative correlation between anti-QS activity in the *Chromobacterium violaceum* CV026 assay and pro-QS activity in the long chain AHL *Pseudomonas aerugionosa* 3-oxo-C12-HSL assay. Collectively, these results suggest the presence of long chain AHLs or homologs in *P. porosa* extracts. In marine systems, long chain AHLs have been detected in sponges [36,65], marine snow [51], and in cultures of coral-associated bacteria [38]. Our results add to the growing evidence that long chain AHLs may be prevalent in marine microbial niches [36,51,52] and could indicate high bacterial densities within or on *P. porosa* tissues. Further study is needed to better understand what role QS stimulatory molecules have in gorgonian microbial ecology.

## 4. Experimental Section

### 4.1. Gorgonian Coral Collection

Fragments of gorgonian corals were collected using SCUBA from La Parguera, Puerto Rico in July of 2007 and June of 2008 at the Looe Key Reef research site (24 33.78′N, 8124.05′W) in the Florida Keys, USA. Gorgonian fragments (5 cm each from 5 individual coral colonies) were collected at depths of 5–10 m. Divers wore gloves to collect the corals and all coral fragments were kept in separate bags. Five colonies were sampled for the following species: *Briareum* sp., *Eunicea laciniata*, *Eunicea tourneforti*, *Plexaura flexuosa*, *Plexaura homomalla*, *Pseudoplexaura porosa*, *Pseudopterogorgia* *americana*, and *Pseudopterogorgia acerosa*, for Puerto Rico, and* Gorgonia ventalina*, *Plexaurella* sp., *Muriceopsis flavida*, and* Eunicea mammosa* for the Florida Keys. All specimens were identified by L.R. Hunt, L.D. Mydlarz, E. Weil (Puerto Rico), and E. Bartells (Florida). Coral fragments were flash frozen in liquid nitrogen and shipped on dry ice to the University of Texas at Arlington, and stored at −80 °C until use.

### 4.2. Extract Preparation

Gorgonian fragments (5 colonies) were pooled to provide sufficient amount of extract for bioassays, then lyophilized on a VirTis Benchtop K lyophilizer (The VirTis Company, Gardiner, NY, USA) and ground in a mortar with a pestle to a fine powder, and subjected to an overnight ethanol extraction at room temperature. 100% ethanol (Decon Labs, Inc., King of Prussia, PA, USA) was added at a ratio of 10 mL to every 0.2 g of homogenized coral. Extracts were then transferred to pre-weighed vials, evaporated to dryness under N_2_, and final weights determined. All samples were diluted in 100% ethanol to a stock concentration of 100 mg/mL and stored at −20 °C until further analysis. 

### 4.3. Antibacterial Assays

Gorgonian ethanol extracts were tested for antimicrobial activity against a suite of Gram-negative and Gram-positive non-marine and marine bacteria (Table 1). The selection of marine bacteria included recognized microbial pathogens of coral such as *Serratia marcescens* PDL100 (courtesy Kim Ritchie), which has been identified as the causative agent of white pox disease in elkhorn coral *Acropora palmata* [66], and *Vibrio alginolyticus* potential pathogen of Caribbean Yellow Band Disease (courtesy Kim Ritchie) [67]. Other marine bacteria included *S. marcescens* from channel water, Cheesequake Salt Marsh, NJ, *V. parahemolyticus* PP-A2 isolated from the mucus of healthy *Pseudoplexaura porosa*,. In addition to marine bacteria, both Gram-positive and Gram-negative tester strains representing many human bacterial pathogens (Table 1) were used to address the range of bacterial inhibition by the gorgonian extracts. Marine bacteria were grown in diluted in nutrient media Difco marine broth (Becton, Dikinson and Co., Le Pont de Claix, France) and non-marine strains in Luria Broth (LB, Miller, Novagen, Merck KGaA, Darmstadt, Germany).

Antimicrobial activity was tested using a bacteria turbidity assay and conducted in 96-well flat-bottom plastic microplates (Greiner bio-one, Monroe, NC, USA). This assay method requires less sample volume than the disc-diffusion assay and has been previously utilized to analyze antimicrobial activity in corals [68]. Assays were run in triplicate wells and experiments contained bacteria with extract, along with the following controls: bacteria only, bacteria + antibiotic, or bacteria + ethanol (to control for ethanol effects). Assays were run at appropriate temperatures for the select bacteria (37 °C for non-marine bacteria and human pathogenic strains, and 29 °C for marine and environmental strains). Ethanol extracts of gorgonians were diluted in nutrient media and 100 µL was added into each well for a final concentration of 250 µg/mL per well (and less than 2.5% ethanol final concentration). Overnight bacteria cultures, in exponential growth, were diluted to an optical density (OD_600_) of 0.2 and 100 µL added to wells containing extracts for a final assay volume of 200 µL per well. The plates were gently mixed and an initial (time 0) OD_600 _reading was recorded with a Biotek Synergy 2 spectrophotometer (Bio-Tek Inc., Winooski VT). Plates were then placed in a shaking incubator and hourly readings were recorded for 5 h with a final reading at 24 h. All assays were run in triplicate or quadruplet. 

Growth inhibition of bacteria cultures was measured by comparing the growth rate (GR) of bacteria with coral extracts to bacteria-ethanol controls. The GR was calculated with the following formula: 3.3 × log ((*t*_f_/*t*_i_)/*n*), where, *t*_f_ = final OD_600_, *t*_i_ = initial OD_600_, and *n* = *t*_f_ − *t*_i_. The linear portion of logarithmic growth was used to select the initial and final hour and was kept standard between runs. Percent growth inhibition was determined by calculating the difference in GR of wells with gorgonian extract to ethanol control values (without the addition of extracts). 

### 4.4. Isolation and Identification of *Vibrio parahaemolyticus*

To isolate culturable bacteria, mucus samples were collected from *Psuedoplexaura porosa* and diluted in sterile seawater. Diluted mucus was then spread onto glycerol artificial seawater agar and incubated at 24 °C. Bacteria that grew on the agar were subcultured into marine broth and identified to species. For species identification of culturable bacteria isolates, bacterial genomic DNA was extracted using a Ultra Clean Microbial DNA isolation kit (Mo BIO Carlsbad, CA, USA) per manufacturer’s instructions. PCR amplification was performed on genomic DNA using Rln (forward primer GCTCAGATTGAACGCTGGCG) and U2 (reverse primer ACATTTCACAACACGAGCAGT) oligonucleotides, which correspond to positions within the *Escherichia coli* 16S rRNA gene [69]. A ~1100 bp product, from PCR amplification, was identified with agarose gel electrophoresis and purified with a PCR purification kit (Qiagen Valencia, CA, USA). PCR products were sequenced via BigDye™ terminator cycling and automated sequencing (ABI Prism 3100xl) using Rln and U2 for forward and reverse strand synthesis respectively. Consensus sequences were then analyzed via GenBank BLAST searches and a percent identity match was made to known bacteria. Thus far, only one bacteria species, *Vibrio parahaemolyticus*, was identified from *P. porosa* mucus and the DNA sequence corresponding to this *V. parahaemolyticus* isolate has been deposited into GenBank (Accession number FJ892748). 

### 4.5. *Pseudomonas aeruginosa* 3-oxo-C12-HSL Bioassay

Long chain *N*-acylhomoserine lactones (AHLs) (*i.e.*, 3-oxo-C12-HSL) were detected in gorgonian ethanol extracts using a sensitive QS reporter strain, *Pseudomonas aeruginosa* PAO1-JP2 (pKR-C12), previously developed [70,71]. The bioassay procedure for detecting long chain AHLs and pro and antiquorum sensing activity with PAO1-JP2 (pKR-C12) is well established [71] and was utilized in the present study with little modification. *P. aeruginosa* was obtained from Professor B. Iglewski from University of Rochester Medical Center, New York, USA. *Pseudomonas aeruginosa* PAO1-JP2 (a las/rhl1 double mutant unable to produce AHL signals) [70] harbors the biosensor plasmid pKR-C12 [72] and was used for testing inhibition or stimulation of QS. The reporter relies on the well-characterized plasmid pKR-C12, which contains a translational fusion of the *las*B elastase gene (a virulence gene) of *P. aeruginosa* to green fluorescent protein (*gfp*) gene, encoding an unstable version of the Gfpmut3 protein [72]. Notably, the unstable variant of *gfp* is useful in that it allows detection of transient gene expression [73] and enables sensitive QS detection in real time. Moreover, pKR-C12 contains the lasR gene, under control of a lac-type promoter, which encodes the cognate receptor for the long chain AHL, 3-oxo-C12-HSL [72]. Thus, the reporter strain PAO1-JP2 (pKR-C12) used in this study was specific to the *las* QS system and most sensitive to long chain AHLs [72]. 

Overnight cultures of the reporter strain grown at 37 °C in LB broth were diluted four-fold into fresh LB media, and grown for an additional 1 h. Crude extracts were added to microtiter plates to a final concentration of 250 µg/mL. After addition of 50 nM of 3-oxododecanoyl-L-homoserine lactone (a synthetic signal molecule, 3-oxo-C12-HSL, Cayman Chemical, Ann Arbor, MI, USA) to reporter strain cultures, 100 µL aliquots of culture were loaded onto plates containing the extracts. For controls, wells were loaded with the same volume of ethanol as the extracts for every experiment. Thereafter, microtiter plates were incubated at 37 °C for 4 h with agitation. Fluorescent signals (excitation = 485 nm; emission = 528 nm) and absorbance (OD_600_) were quantified with a Synergy 2 spectrophotometer (Bio-Tek Instruments, Gen 5 Software, Winooski, VT, USA). Values were reported as % inhibition fluorescence relative to control values (minor fluorescence due to ethanol without the addition of extracts). To test for stimulation of QS, assay conditions were the same as above except the AHL signaling molecule (3-oxo-C12-HSL) was not added to the reporter strain culture. Stimulatory values were reported as relative fluorescent units (RFU) and were compared to negative control cultures (no AHL added). 

### 4.6. *Chromobacterium violaceum* CV026 Bioassay

The *N*-acylhomoserine lactones (AHL) biosensor strain *Chromobacterium violaceum* CV026 served as an indicator organism by quantifying violacein (pigment) synthesis, a phenotype under QS control. Violacein production is dependent upon external addition of N-hexanoylhomoserine lactone (C6-HHL, Cayman Chemical, Ann Arbor, MI, USA) [64] in *C. violaceum* CV026, QS induction was tested by adding coral extracts to a 96-well plates containing overnight (18 h) cultures of *C.violaceum* CV026 following methods previously described [63]. Plates were incubated for 16 h at 27 °C on a plate shaker. Violacein pigment production was detected by drying the plates at 60 °C overnight, resolubilizing the violacein pigment in 100 µL of DMSO, and determining *A*_590_. HHL was added to assay wells at an optimal induction concentration (3.7 × 10^−8^ M HHL) to test for QS inhibition. The percentage inhibition of violacein production was correlated to ethanol control values without the addition of extracts and values were reported as percent inhibition relative to control values. For induction of QS, HHL was not added to assay wells and the presence of violacein in the wells indicated QS induction. For controls, wells were loaded with the same volume of ethanol as extracts for every experiment. 

### 4.7. Statistical Analysis

Data were tested for normality (Shapiro-Wilk) and homogeneity of variances (Levene) prior to analysis. Data sets met parametric analyses constraints and were analyzed using ANOVA with Tukey post-hoc tests with JMP Statistical Discovery Software version 9.0 (SAS Institute Inc., Cary, NC, USA). 

## 5. Conclusions

In this study, we report evidence for both inhibition and stimulation of quorum sensing (QS) activity and strong antimicrobial activity against select bacteria in Caribbean gorgonian extracts. These data indicate that microbial regulation in corals may occur by several different mechanisms, such as controlling bacteria growth directly or indirectly through modulating QS. Since the coral extracts were not very effective against several representative marine bacteria, controlling bacteria through direct inhibition or induction of the QS signals may be an alternative strategy. Although not a strategy employed equally by all genera of corals examined in this study. To our knowledge, this is the first demonstration of QS stimulation in Caribbean gorgonian corals. Testing of chemical extracts can suggest the presence of AHLs (or compounds with similar activity) in coral tissues, but cannot determine which component of the holobiont are the source of the AHL-like molecules. Identification of the AHL source is a crucial next step, as well as the bacterial phenotypes under AHL control. Our study also found that Caribbean gorgonians have strong antimicrobial activity against pathogenic bacteria of human concern and little antimicrobial activity against certain marine bacteria. Gorgonians may need to rely on other mechanisms, such as QS or inducible immune defenses, to control these relevant bacteria. Further work is needed to better understand microbial regulation in the coral environment and further characterize gorgonian antimicrobials with potential benefit to humans.
